# Co-transcriptional histone methylations

**DOI:** 10.1186/1756-8935-6-S1-O28

**Published:** 2013-03-18

**Authors:** Stephen Buratowski

**Affiliations:** 1 Department of Biological Chemistry and Molecular Pharmacology, Harvard Medical School, Boston, MA 02115, USA

## 

Genes transcribed by RNA polymerase II show a stereotypical pattern of histone H3 methylations. H3K4 trimethylation peaks near the transcription start site, with dimethylation peaking further downstream. H3K36 methylation levels increase with distance from the promoter. It is not yet completely understood how this pattern is generated, but the mechanism involves direct interactions between the transcribing polymerase and the methyltransferases Set1/COMPASS (for H3K4) and Set2 (for H3K36). We are working towards a better understanding of how this coupling works. We are also very interested in the function of these co-transcriptional histone modifications. Previous work has shown that methylated H3K36 recruits the histone deacetylase complex Rpd3S and that H3K4me2 recruits the Set3C histone deacetylase complex. Therefore, a major function of co-transcriptional histone methylation is to reset transcribed chromatin to a state less conducive to nucleosome turnover. This pathway represses cryptic promoters inside the transcribed gene, but also slows transcription elongation through this region. We and others have found that these histone methylation-deacetylation pathways can be targeted via overlapping non-coding transcription to regulate nearby promoters. In contrast to H3K4me2 and H3K36me2/3, H3K4me3 correlates with high turnover nucleosomes near transcription start sites. Available data suggests that H3K4me3 is recognized by three complexes containing ING/Yng proteins via their PHD finger domains. The yeast proteins Yng1 and Yng2 (as well as their metazoan counterparts) are subunits of histone acetyltransferase complexes, suggesting that H3K4me3 can target histone acetylation. Since acetylation is correlated with nucleosome remodeling, this putative pathway could be important for gene activation. Surprisingly, the third Yng protein (Pho23, as well as its metazoan counterparts) is a subunit of the Rpd3L histone deacetylase complex, hinting at a more complex series or cycle of acetylation and deacetylation. We are continuing to probe the functions of these pathways and some of our latest work will be presented.

